# Vinylene‐Linked Covalent Organic Frameworks by Base‐Catalyzed Aldol Condensation

**DOI:** 10.1002/anie.201905886

**Published:** 2019-09-09

**Authors:** Amitava Acharjya, Pradip Pachfule, Jérôme Roeser, Franz‐Josef Schmitt, Arne Thomas

**Affiliations:** ^1^ Department of Chemistry-Functional Materials Technische Universität Berlin Hardenbergstr. 40 BA2, 10623 Berlin Germany

**Keywords:** covalent organic frameworks, [2+2] cycloaddition, π⋅⋅⋅π stacking, porous polymers, vinylene links

## Abstract

Two 2D covalent organic frameworks (COFs) linked by vinylene (−CH=CH−) groups (V‐COF‐1 and V‐COF‐2) are synthesized by exploiting the electron deficient nature of the aromatic *s*‐triazine unit of *C_3_*‐symmetric 2,4,6‐trimethyl‐*s*‐triazine (TMT). The acidic terminal methyl hydrogens of TMT can easily be abstracted by a base, resulting in a stabilized carbanion, which further undergoes aldol condensation with multitopic aryl aldehydes to be reticulated into extended crystalline frameworks (V‐COFs). Both V‐COF‐1 (with terepthalaldehyde (TA)) and V‐COF‐2 (with 1,3,5‐tris(*p*‐formylphenyl)benzene (TFPB)) are polycrystalline and exhibit permanent porosity and BET surface areas of 1341 m^2^ g^−1^ and 627 m^2^ g^−1^, respectively. Owing to the close proximity (3.52 Å) of the pre‐organized vinylene linkages within adjacent 2D layers stacked in eclipsed fashion, [2+2] photo‐cycloadditon in V‐COF‐1 formed covalent crosslinks between the COF layers.

## Graphical abstract

**Come on over vinylene**: The synthesis of two vinylene‐linked (−CH=CH−) covalent organic frameworks (COFs) by base‐catalyzed aldol condensation is reported. Structural properties of the highly crystalline COF were investigated.

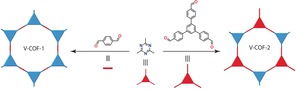

The discovery of covalent organic frameworks (COFs) demonstrated that organic building blocks could be reticulated via strong covalent bonds into well‐ordered 2D and 3D extended crystalline frameworks.^1^, ^2^, ^3^, ^4^, ^5^, ^6^ These ordered crystalline materials feature defined porosities and functionalities and have consequently garnered increasing attention in various applications such as gas storage and separation,^7^, ^8^ energy storage,^9^, ^10^ photovoltaics,^9^, ^11^ opto‐electronics,^12^, ^13^ proton and ion conduction,^14^, ^15^, ^16^, ^17^, ^18^, ^19^ and heterogeneous catalysis.^20^, ^21^, ^22^, ^23^, ^24^, ^25^, ^26^ The formation of such extended crystalline frameworks is generally achieved by implementing reversible condensation reactions to polymerize rigid building blocks. Careful control over the rate of reversible bond formation allow the construction of the desired crystalline framework by applying the principles of reticular chemistry.^27^, ^28^, ^29^ Until recently, linkages (that is, bonds formed to reticulate the building blocks) reported for COF synthesis were mostly based on reversible B−O,^1^, ^2^, ^16^, ^30^, ^31^ B−N,^32^ Si−O,^33^, ^34^ and C−N bond formation.^35^, ^36^, ^37^, ^38^, ^39^ The reversible nature of those linkages affected by the insertion–deletion of water molecules, allow the formation of the desired extended crystalline frameworks, but was also identified to be detrimental in terms of chemical stability, especially in aqueous media.^28^, ^40^ Indeed, although thermally robust, many COFs are prone to hydrolysis and structural collapse in aqueous/acidic media or even in contact with moisture. Several strategies, including keto–enol tautomerization,^35^ interlayer stacking optimization,^36^ or post‐functionalization^41^, ^42^ have been applied to circumvent these limitations, yielding COFs featuring higher chemical and thermal stability.

In this context, recently reported 2D COFs based on cyanovinylene (−CH=C(CN)−) linkages hold the promise for the generation of chemically stable COFs as the cyanovinylene linkage is significantly less prone to hydrolysis compared to boroxine, boronate ester, or imine linkages that are mostly used for the synthesis of COFs.^10^, ^43^ The challenge to crystallize a COF by reversible −CH=C(CN)− bond formation was overcome by implementing reversible Knoevenagel condensation of 1,4‐phenylenediacetonitrile (PDAN) with multitopic aldehyde‐functionalized building blocks. Such COFs featuring fully conjugated backbones are also promising materials in terms of optoelectronic or photovoltaic applications.^44^

Herein, we report the synthesis of two purely vinylene (−CH=CH−) linked 2D COFs (V‐COF‐1 and V‐COF‐2) achieved by base‐catalyzed reversible aldol condensation, exploiting the highly electron deficient s‐triazine core of 2,4,6‐trimethyl‐*s*‐triazine (TMT). The methyl protons of TMT are acidic and undergo base‐catalyzed aldol condensation with benzaldehyde to yield 2,4,6‐tristyryl triazine quantitatively (Scheme 1 a).^45^, ^46^ V‐COF‐1 and V‐COF‐2 were obtained by extending this model reaction to terepthalaldehyde (TA) and 1,3,5‐tris(4‐formyl)phenyl benzene (TFPB) (Scheme 1 b). During our investigation on the base‐catalyzed pathway, Yaghi et al. reported the acid‐catalyzed synthesis of a biphenyl analogue of V‐COF‐1, which however could not be synthesized after extensive trials employing basic conditions.^47^ On the other hand, we were so far also unable to synthesize crystalline V‐COF‐1 and ‐2 using the reported acid‐catalyzed pathway, showing that the choice of reaction conditions can be crucial to obtain V‐COFs with varying backbone structure. V‐COF‐1 and V‐COF‐2, possess surface areas (SA_BET_) of 1341 m^2^ g^−1^ and 627 m^2^ g^−1^, respectively. Although chemically and thermally stable, we identified that V‐COF‐1 is photo‐responsive, as the pre‐organized vinylene groups of adjacent 2D layers offer a platform for [2+2] cycloaddition upon UV/Vis irradiation fulfilling Schmidt's criteria for photocyclization.^48^

**Scheme 1:**
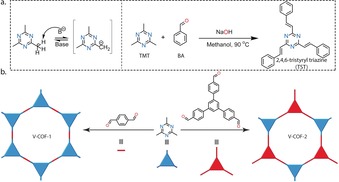
V‐COF synthesis. a) Illustration of the base‐catalyzed aldol condensation of 2,4,6‐trimethyl‐s‐triazine (TMT) and benzaldehyde (BA) to yield 2,4,6 tristylryl‐s‐triazine (TST). b) Representation of reticulation of the crystalline V‐COFs by condensation of 2,4,6 trimethyl‐*s*‐triazine with ditopic or tritopic aldehydes, respectively.

Syntheses of V‐COF‐1 and V‐COF‐2 were performed by solvothermal condensation of TMT with TA and TFPB, in 7:1 and 1:1 (methanol/mesitylene) solvent mixtures, respectively, for 4 days at 180 °C in presence of NaOH as base (Figure 1 a). Both the COFs were collected as light‐yellow polycrystalline solids by filtration and further washed with methanol, water, and acetone before being dried under vacuum at 100 °C overnight.

**Figure 1:**
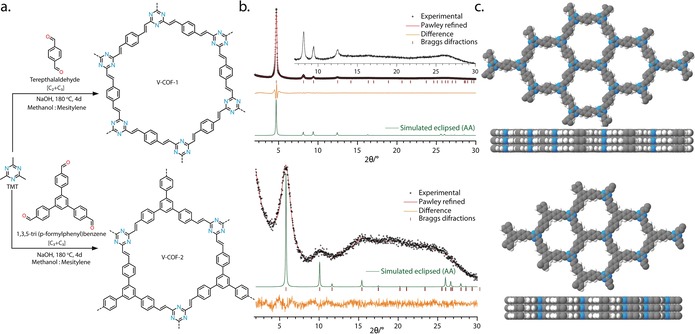
Synthesis and structural characterization of V‐COFs. a) Synthetic conditions for the reticulation of V‐COF‐1 and V‐COF‐2. b) PXRD patterns of V‐COF‐1 and V‐COF‐2: experimental patterns (black), Pawley refined profile curves (red), Bragg diffractions (brown), difference (orange), simulated patterns for the eclipsed layer stackings (green), and c) structural models of both V‐COF‐1 and V‐COF‐2 with their respective stacking patterns.

Crystallinity of V‐COF‐1 and V‐COF‐2 was assessed by powder X‐ray diffraction (PXRD) analyses. The experimental PXRD pattern of V‐COF‐1 and V‐COF‐2 confirmed the formation of a crystalline framework with no evidence of remaining starting material (Supporting Information, Figure S2). Given the connectivity of the building blocks, several models with different stacking modes were generated in hexagonal 2D nets with **hcb** topology (Supporting Information, Section S4). A good match was found between the experimentally obtained PXRD patterns and the calculated patterns of a fully eclipsed model (Figure 1; Supporting Information, Figures S3, S5). The final lattice parameters were extracted after Pawley refinement and V‐COF‐1 was found to crystallize in a hexagonal unit cell (*P*6/*m*, *a*=*b*=21.6934 Å, *c*=3.5232 Å, *R_P_*=2.66 %; *R_WP_*=3.40 %) with similar unit cell parameters as the isoreticular LZU‐1 constructed from benzene nodes and imine linkages.^49^ V‐COF‐2 was poorly crystalline as evidenced by the broad diffraction peaks observed experimentally, but was found to crystallize in a similar hexagonal unit cell (space group: *P* $\bar{6}$ , *a*=*b*=17.5632 Å, *c*=3.4544 Å, *R*_p_=2.82 %; *R_wp_*=3.58 %) (Figure 1; Supporting Information, Figures S4, S6).

Fourier‐transform infrared (FTIR) spectroscopy analyses were applied to assess the structural integrity of the crystalline framework (Figure 2 a). Complete disappearance of −C=O− stretching frequency of starting aldehyde monomers at 1690 cm^−1^ and appearance of a new band at 1631 cm^−1^ attributed to −C=C− stretching indicated complete condensation of the starting building blocks and the successful formation of the vinylene linkage in V‐COF‐1 and V‐COF‐2.

**Figure 2:**
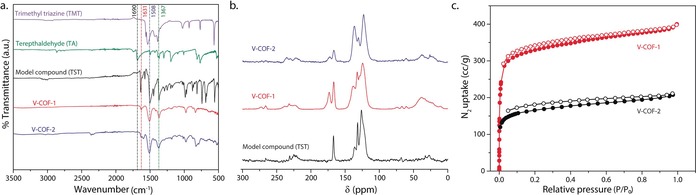
Structural characterization of V‐COFs. a) FTIR analyses of V‐COFs compared with the starting materials (TMT and TA) and the model compound (TST). b) ^13^C CP‐MAS NMR spectra of V‐COF‐1 and V‐COF‐2 and model compound (TST). c) N_2_ sorption measurements of V‐COF‐1 and V‐COF‐2 powders.

^13^C cross‐polarization magic‐angle‐spinning (CP‐MAS) NMR spectroscopy confirmed the full conversion of starting monomers as no residual carbonyl resonance located at δ≈190 ppm could be detected. The two expected signals for the vinylene (−C=C−) carbons at about 138.1 ppm and 132.1 ppm could be unambiguously assigned within the aromatic carbon signals (Figure 2 b; Supporting Information, Figure S7). V‐COF‐1 and V‐COF‐2 surprisingly exhibited two distinct signals for the aromatic triazine carbon atoms at about 172.9 and about 166.4 ppm in the ^13^C CP‐MAS NMR spectra, respectively, while just one peak, as expected, was observed for the molecular model compounds. Splitting of the triazine signals has been also observed in the solid‐state ^13^C NMR spectrum of melamine and was attributed to different H‐bonding environment leading to two chemically inequivalent carbon atoms.^50^, ^51^ However, this explanation could be ruled out here, since there is no electron‐deficient hydrogen donor within the building blocks. Instead, the second triazine environment is probably caused by a [2+2] cycloaddition between vinylene groups.^52^, ^53^, ^54^ This is corroborated by the presence of a broad signal in the aliphatic region (δ≈38.1 ppm) indicating the formation of cyclobutane moieties in the COFs (see below).

The permanent porosity of V‐COF‐1 and V‐COF‐2 was evaluated by low‐pressure nitrogen (N_2_) and argon (Ar) sorption studies on evacuated samples, at 77 K and 87 K, respectively. A steep gas uptake in the low relative pressure range (*p*/*p*_0_<0.05) of the N_2_ adsorption branch shows the microporosity of the samples (Figure 2 c). The Brunauer–Emmett–Teller surface areas (SA_BET_) calculated from the N_2_ adsorption were found to be 1341 m^2^ g^−1^ and 627 m g^−1^, respectively. The SA_BET_ of V‐COF‐1 is almost twice as high as the SA_BET_ reported for the isoreticular LZU‐1 COF (729 m^2^ g^−1^).^49^, ^55^ The corresponding pore size distribution of 1.69 nm was derived by fitting the Ar adsorption branch data at 87 K with a cylindrical quenched‐solid density functional theory (QSDFT) model, and matched well with the theoretically calculated pore size of 1.6 nm of the proposed eclipsed structural model (Supporting Information, Figure S8). The pore size distribution of V‐COF‐2 was found to be centered at 0.82 nm, derived by fitting the N_2_ adsorption branch data at 77 K with the QSDFT model and again matched well with theoretically calculated pore size of 0.8 nm (Supporting Information, Figure S9).

Architectural stability of the V‐COF‐1 and V‐COF‐2 was investigated by thermogravimetric analysis (TGA) under N_2_ atmosphere (Supporting Information, Figures S10, S11). After an initial weight loss of about 2 wt % owing to desorption of adsorbed solvents below 100 °C, the frameworks remained stable until 400 °C. Chemical stability of crystalline V‐COF‐1 and V‐COF‐2 was further investigated in harsh acidic and basic conditions (Supporting Information, Figures S12, S13). Both V‐COF‐1 and V‐COF‐2 were found to be stable in concentrated acidic and basic medium for at least 4 days. V‐COF‐1 was also found to be stable in common organic solvents such as THF, acetone, and DMF, for 4 days, with no signs of decomposition (Supporting Information, Figure S14).

Considering the high crystallinity and conjugated structure, we investigated the optical properties of V‐COF‐1. The diffuse‐reflectance UV/Vis (UV‐DRS) spectrum of V‐COF‐1 shows a distinct red‐shift in absorbance with respect to the monomers (TA and TMT), indicating a higher degree of conjugation (Supporting Information, Figure S15). V‐COF‐1 shows an absorbance edge at about 410 nm, whereas the absorbance tail is extended up to about 535 nm. Interestingly, V‐COF‐1 was found to be photosensitive, as the light‐yellow color of pristine COF powders slowly faded with time upon sunlight irradiation, indicating a structural change within V‐COF‐1 (Figure 3 a). To further elucidate the effect of light irradiation, V‐COF‐1 powder was irradiated directly with UV/Vis light (*λ*≈320–500 nm) considering that the maximum absorbance of the COF lies within this wavelength range (Supporting Information, Section S10). ^13^C CP‐MAS NMR spectra of the irradiated (48 h) sample revealed a cycloaddition reaction between the COF layers. Thus, the vinylene carbon signal (δ≈138.1, 132.4 ppm) intensity significantly decreased and the ratio of the triazine carbon signals (at δ≈172.9 and δ≈166.4) reversed compared to the pristine COF (Figure 3 b). Finally, a broad signal (δ≈38.1 ppm) at the aliphatic region of the ^13^C CP‐MAS NMR spectra proved the formation of cyclobutane moieties.

**Figure 3:**
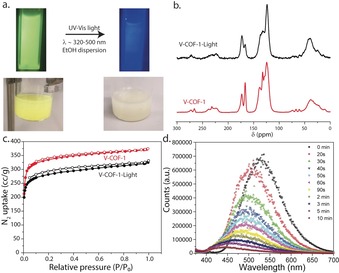
Effect of light irradiation on V‐COF‐1. a) Photographs of V‐COF‐1 dispersions, and b) ^13^C CP‐MAS NMR spectra and c) N_2_ sorption isotherms of V‐COF‐1 before (V‐COF‐1) and after light irradiation (V‐COF‐1‐Light). d) Fluorescence emission quenching upon irradiation of light with time, monitored by fluorescence emission spectroscopy.

Solid‐state supramolecular photochemistry offers an efficient way to engineer new functional polymers by taking advantage of the preorganization of reactive moieties in the crystalline matrix.^56^, ^57^, ^58^ In general, different strategies such as hydrogen bonding interaction, π⋅⋅⋅π stacking, and metal–ligand interactions have been explored to control the topochemical arrangement of the reactive modules. Efficient π⋅⋅⋅π stacking of 2D layers within 2D COFs offer a suitable platform for the preorganization of photoreactive moieties in a particular fashion to undergo photochemical reactions.^59^ Topochemical [2+2] photocycloaddition in the solid state requires parallel orientation of two double bonds within a distance of 3.5–4.2 Å, entailed by Schmidt's criteria.^48^ Thus, preorganization of the vinylene based templates are necessary for controlled synthesis of new 2D or 3D polymers. Schlüter et al. reported the synthesis of a 2D polymer by topochemical [2+2] photocycloaddition of a trivinyleneic monomer, whereas numerous vinyleneic building units inside metal organic frameworks (MOFs) or coordination polymers are also reported to undergo [2+2] cycloaddition.^60^, ^61^ Considering the high crystallinity and eclipsed stacking of 2D layers, V‐COF‐1 offers a suitable platform for [2+2] photo‐cycloaddition within the vinylene linkages of adjacent layers. Still, this is the first report where parallel oriented vinylene moieties of adjacent layers undergo [2+2] cycloaddition within the 2D layers of a covalent organic framework.

The effect of cyclobutane formation from the vinylene linkages was also observed in the fluorescence emission spectrum of the V‐COF‐1 powders measured over time with light irradiation (Figure 3 d). The pristine COF powders dispersed in ethanol was found to be highly fluorescent upon excitation at *λ*≈365 nm. With light irradiation over time, a fast quenching of fluorescence emission together with the blue‐shift of the emission maximum was observed and fluorescence emission was completely quenched after 10 minutes. This can be expected, as the degree of conjugation decreases upon formation of cyclobutane from conjugated vinylenes.^62^

Crystallinity of V‐COF‐1 was affected upon the [2+2] photo‐cycloaddition, as sp^2^‐hybridized vinylene carbons convert into sp^3^‐cyclobutane species, causing severe strain within the crystalline lattice (Figure 4). To compensate the strain within the 2D layers, V‐COF‐1 framework renders amorphous (Supporting Information, Figure S17). Such a change in crystallinity upon [2+2] photocycloaddition within crystalline lattices has been widely investigated in vinylene‐based monomer single crystals, sometimes with a photosalient effect.^63^ Notably, the framework remained porous even after exposure to UV/Vis irradiation for long time (48 h) with a SA_BET_ of 1093 m^2^ g^−1^ (Figure 3 c).

**Figure 4:**
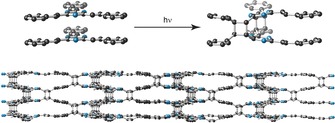
Illustration of the [2+2] photocycloaddition between the layers and possible structural changes in the V‐COF‐1 lattice (side view). Note that the partial cycloaddition of vinylenes is represented, corroborating the ^13^C CP‐MAS NMR spectrum of V‐COF‐1 after light irradiation.

In summary, we report the successful crystallization of two new vinylene‐linked 2D COFs (V‐COF‐1 and V‐COF‐2), achieved by base‐catalyzed aldol condensation of TMT with ditopic terepthalaldehyde (TA) and tritopic 1,3,5‐tris(p‐formylphenyl)benzene (TFPB). This work demonstrates a successful strategy to exploit the highly electron‐deficient s‐triazine core for the crystallization of 2D vinylene‐linked COFs at base‐catalyzed conditions. Both COFs were found to be chemically stable under concentrated acidic as well as in basic conditions. [2+2] photocycloaddition within the columnar π⋅⋅⋅π stacked 2D layers of a COF was observed for the first time upon UV/Vis light irradiation on V‐COF‐1 powders. Loss of crystallinity of V‐COF‐1 was observed after light irradiation owing to formation of highly strained cyclobutane rings within the 2D layers, as confirmed by a ^13^C CP‐MAS NMR measurments. However, the framework remains porous with a SA_BET_ of 1093 m^2^ g^−1^ even after exposure to UV/Vis irradiation for long time (48 h). Therefore, our focus is now dedicated towards the cycloreversion of the cyclobutanes, which could lead to a photo‐switchable crystalline framework and could be implemented as a rewritable memory storage device. However, such a task is extremely difficult to achieve in the solid polycrystalline state, as the [2+2] cycloreversion from cyclobutane to parent vinylenes can follow different pathways. Thus, suitable conditions to photoswitch the frameworks reversibly are currently under investigation.

## Conflict of interest

The authors declare no conflict of interest.

## Supporting information

As a service to our authors and readers, this journal provides supporting information supplied by the authors. Such materials are peer reviewed and may be re‐organized for online delivery, but are not copy‐edited or typeset. Technical support issues arising from supporting information (other than missing files) should be addressed to the authors.

Supplementary
