# Supplementary material for: Vinylene‐Linked Covalent Organic Frameworks by Base‐Catalyzed Aldol Condensation
Source: Angew Chem Int Ed Engl. 2019 Sep 9;58(42):14865–70. doi: 10.1002/anie.201905886 (PMC6851556; doi:10.1002/anie.201905886)
Supplement: Supplementary file 1 — Supplementary [file ANIE-58-14865-s001.pdf]

Angewandte Chemie
Eine Zeitschrift der Gesellschaft Deutscher Chemiker

Supporting Information

# Vinylene-Linked Covalent Organic Frameworks by Base-Catalyzed Aldol Condensation

*Amitava Acharjya, Pradip Pachfule, Jérôme Roeser, Franz-Josef Schmitt, and Arne Thomas**

anie_201905886_sm_miscellaneous_information.pdf

# Table of contents

**Section S1:** Materials and general methods………………………………………………... 3

**Section S2:** Complete synthetic procedure………………………………………………… 5

**Section S3:** PXRD analysis confirming V-COF-1 Crystallization…………………………..7

**Section S4:** Structure Modeling and Atomic Coordinates of V-COFs……………………..8

**Section S5:** NMR characterization of model compound…………………………………...14

**Section S6:** Pore size distribution analysis of V-COFs…...……………………………….15

**Section S7:** Thermo-gravimetric (TGA) analysis of V-COFs..…………………………….16

**Section S8:** Stability test of V-COF-1 and V-COF-2………………………………………..17

**Section S9:** Optical properties of V-COF-1…………………………………………………19

**Section S10:** [2+2]-photocycloaddition……………………..…………………………….20

**References:** ……………………………………………………………………………….. 21

## Section S1. Materials, general instruments and methods:

***Materials:***

All the reagents and solvents used for the synthesis were commercially available and used without further purification. The anhydrous methanol (99.9 %) and mesitylene (1,3,5-trimethylbenzene, > 98%) were purchased from Sigma Aldrich Chemicals. The precursors such as ethyl acitimidate hydrochloride (abcr, 99%) and terepthalaldehyde (sigma, 99%) were purchased and used as received. 1,3,5-tris(4-formylphenylphenyl)benzene (TFPB) was synthesized following reported procedure[1].

***General instrumentation and methods:***

The autoclaves (23 mL) used for COF synthesis were from Parr instrument compay.

***Thermo-gravimetric Analysis (TGA):***

Thermo-gravimetric analyses (TGA) were carried out on a Mettler Toledo TGA/DSC1 Star System analyzer under nitrogen atmosphere at a heating rate of 5 °C $min^{-1}$ within the temperature range of 25–800 °C.

***Powder X-ray diffraction (PXRD) analysis:***

Powder X-ray diffraction data was collected on a Bruker D8 Advance diffractometer in reflection geometry operating with a Cu Kα anode (λ = 1.54178 Å) operating at 40 kV and 40 mA. Samples were ground and mounted as loose powders onto a Si sample holder. PXRD patterns were collected from 2 to 60 2$\theta$ degrees with a step size of 0.02 degrees and an exposure time of 2 seconds per step.

***Fourier transform infrared spectroscopy (FT-IR) analyses:***

The Fourier transform infrared spectroscopy (FTIR) analyses of the samples were carried on Varian 640IR spectrometer equipped with an ATR cell.

***NMR measurements:***

$^{1}H$ NMR for the samples dissolved in suitable solvents were carried on Bruker Avance II 200. $^{13}C$ Solid-state NMR (cross polarization magic-angle spinning (CP/MAS)) spectra were carried out on a Bruker Avance 400 MHz spectrometer operating at 100.6 MHz.

***Solid-state diffuse reflectance Ultraviolet–visible spectroscopy (UV-DRS) analysis:***

Solid-state diffuse reflectance Ultraviolet–visible spectroscopy (UV-vis) spectra of the as pristine COF powders and starting monomers have been collected on Varian Cary 300 UV-Vis Spectrophotometer.

***Physisorption measurements:***

$N_2$ and Ar sorption measurements were performed on a volumetric sorption instrument (Autosorb–iQ–MP). Prior to the gas sorption studies of COFs, the samples were dried under a dynamic vacuum ($<10^{-3}$ Torr) at room temperature (RT) followed by heating 150 °C for 24 h. Using the Ar adsorption isotherms, the surface areas were calculated over a pressure range 0.01–0.9 = $p/p_0$ using Brunauer-Emmett-Teller (BET), and pore size distributions were calculated using the quenched solid density functional theory (QSDFT) method on the Ar adsorption branch.

***Fluorescence Emission Spectroscopy:***

Fluorescence emission was measured with a Fluoromax-2 spectro-fluorometer (Horiba Jobin Yvon, Bensheim, Germany). Slits were set to 4 nm for excitation and 2 nm for emission, while the integration time was 0.5 s and the increment 1 nm. The sample was excited at 365 nm, and emission spectra were recorded in a suitable range centred around the emission maximum between 370 and 700 nm.

**Section S2. Synthesis of organic linker, model compound and V-COFs:**

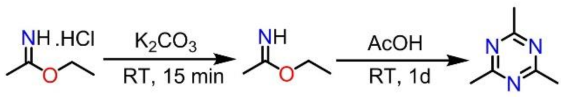


**Scheme S1.** Synthetic scheme for the preparation of 2,4,6 trimethyl *s*-triazine.

**Synthesis of 2,4,6 trimethyl *s*-triazine:** The synthesis of 2,4,6 trimethyl *s*-triazine was adapted from a reported procedure.[2] To prepare the free base, 17.28 g (0.14 moles) of ethyl acitimidate hydrochloride was added quickly at 20-25 °C to a vigorously stirred mixture of 40 mL of methylene dichloride and 31.8g (0.23 moles) of potassium carbonate saturated solution in water. After 10 min., the organic phase was collected and the aqueous phase was extracted 2-3 times with 50 mL dichloromethane. All the extracts were combined and dried over anhydrous sodium sulphate. The solution was filtered and then stripped of solvents on a rotary evaporator to a concentrated solution. To this solution 1.8 mL of glacial acetic acid was added slowly (20-30 min) and the temperature was held at 25 °C for 1 hour. The mixture was then allowed to stand for 1 day. The reaction mixture was then stripped of solvents and the residue was diluted by adding dichloromethane. The solution was filtered from crystalized acetamidine acetate and residual acetic acid was neutralized with a concentrated aqueous potassium carbonate and dried with sodium sulphate. The solution was then stripped of most of the solvent and then kept at room temperature for the evaporation of the rest to finally get a solid. After solvent evaporation colourless crystals of 2,4,6 trimethyl *s*-triazine were obtained after purification by sublimation. Sublimated crystals were also submitted to determine the single crystal x-ray structure.

**$^1$H NMR (400 MHz, $CDCl_3$):** δ [ppm] = 2.53 (s, 9H, $CH_3$).

**$^{13}$C NMR (100 MHz, $CDCl_3$):** δ [ppm] = 175.9, 25.5.

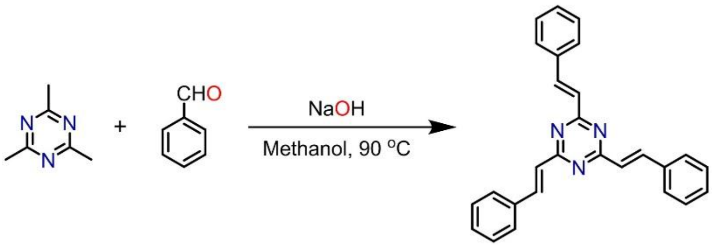


**Scheme S2.** Synthetic scheme for the preparation of 2,4,6 tristyryl *s*-triazine (model compound).

**Synthesis of 2,4,6 tristyryl *s*-triazine (model compound):** the synthesis of 2,4,6 tristyryl s-triazine (TST) was adapted from a reported procedure.[3] A 25 mL round bottom flask was charged with 61.5 mg (0.5 mmol) of 2,4,6 trimethyl *s*-triazine which was dissolved in 5 mL methanolic KOH solution (84 mg KOH in 5mL). A solution of 212.2 mg (2 mmol) of benzaldehyde in methanol (5mL) was then added. The mixture was kept under reflux at 90 °C for 1 day. A white precipitate was obtained which was filtered off, washed with methanol and dried at 80 °C. Single crystals of the compound was obtained from a saturated solution of the model compound in chloroform.

**Yield: 98%**

**$^{1}$H NMR (400 MHz, $CDCl_3$):** δ [ppm] = 8.30 (d, J=15.9 Hz, 3H), 7.73–7.68 (m, 6H), 7.48–7.37 (m, 9H), 7.19 (d, J= 15.9 Hz, 3H)

**$^{13}$C NMR (100 MHz, $CDCl_3$):** δ [ppm] = 171.0, 142.2, 135.4, 130.06, 128.9, 128.2, 125.8

**Synthesis of V-COF-1:** A teflon-lined steel autoclave (23 mL) was charged with 2,4,6 trimethyl *s*-triazine (TMT) (24.6 mg, 0.2 mmol), terepthalaldehyde (TA) (40.23 mg, 0.3 mmol), NaOH (35 mg), methanol (14mL) and mesitylene (2mL). The autoclave was sealed and placed in a preheated oven at 180 °C for 4 days. After four days, the autoclave was cooled down and the formed precipitate was collected by filtration and thoroughly washed with water, methanol and acetone. V-COF-1 was obtained as a (fluffy) yellow powder after a further drying step under vacuum at 100 °C.

***Isolated yield:*** *83% (45 mg)*

**Synthesis of V-COF-2:** A teflon-lined steel autoclave (23 mL) was charged with 2,4,6 trimethyl s-triazine (TMT) (19.68 mg, 0.16 mmol), 1,3,5-tris(4-formylphenyl)benzene (TFPB) (62.46 mg, 0.16 mmol), NaOH (30 mg), methanol (8mL) and mesitylene (8mL). The autoclave was sealed and placed in a preheated oven at 180 °C for 4 days. After four days, the autoclave was cooled down and the formed precipitate was collected by filtration and thoroughly washed with water, methanol and acetone. V-COF-2 was obtained as a (fluffy) yellow powder after a further drying step under vacuum at 100 °C.

***Isolated yield:*** *82% (60.2 mg)*

**Section S3. PXRD analysis confirming V-COF-1 crystallization:**

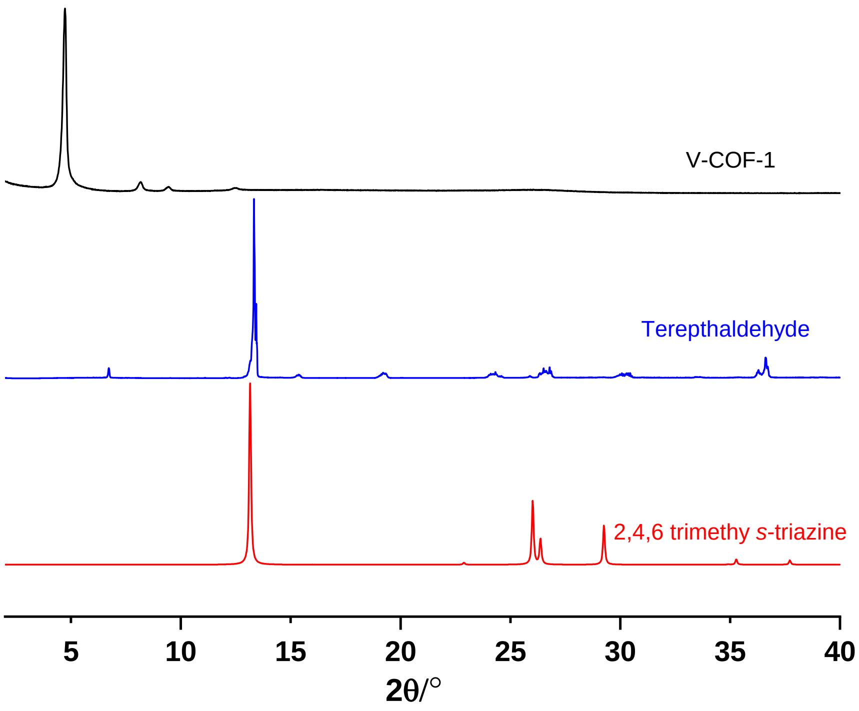


**Figure S2**. Experimental PXRD patterns of V-COF-1 (black curve), terephalaldehyde (blue curve) and 2,4,6 trimethyl *s*-triazine (red curve).

**Section S4. Structure Modeling and Atomic Coordinates of V-COFs:**

Structure models were generated using the *Material Studio Modelling* 5.0 package.[4] Geometry optimization was performed using the Forcite module and UFF forcefield.[5] Full profile pattern fitting (Pawley) was performed against the experimental powder pattern using the *Reflex* module.

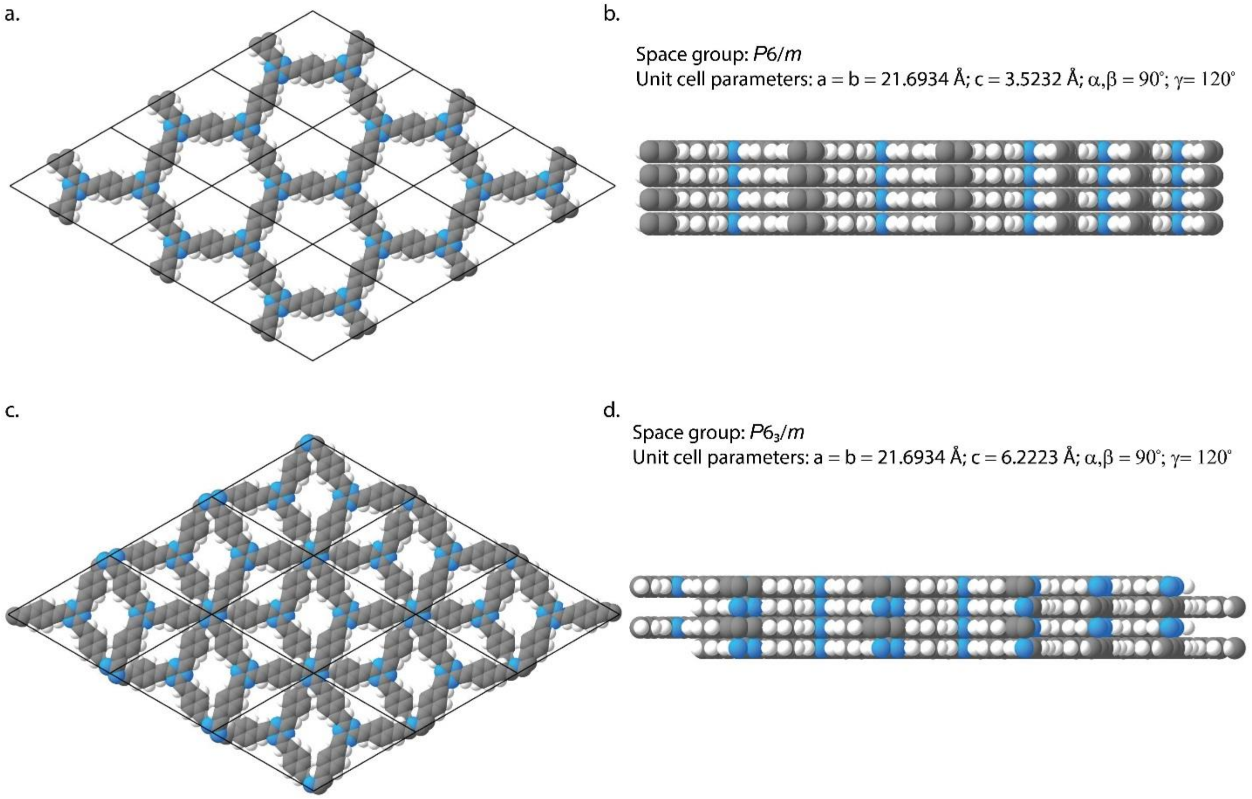


**Figure S3.** Structural models of V-COF-1. a) Theoretical structure of V-COF-1 with eclipsed (AA) stacking arrangement. b) Side view of V-COF-1 structure with eclipsed stacking arrangement c) The theoretical structure of V-COF-1 with staggered (AB) stacking arrangement. d) Side view of V-COF-1 structure with staggered stacking arrangement.

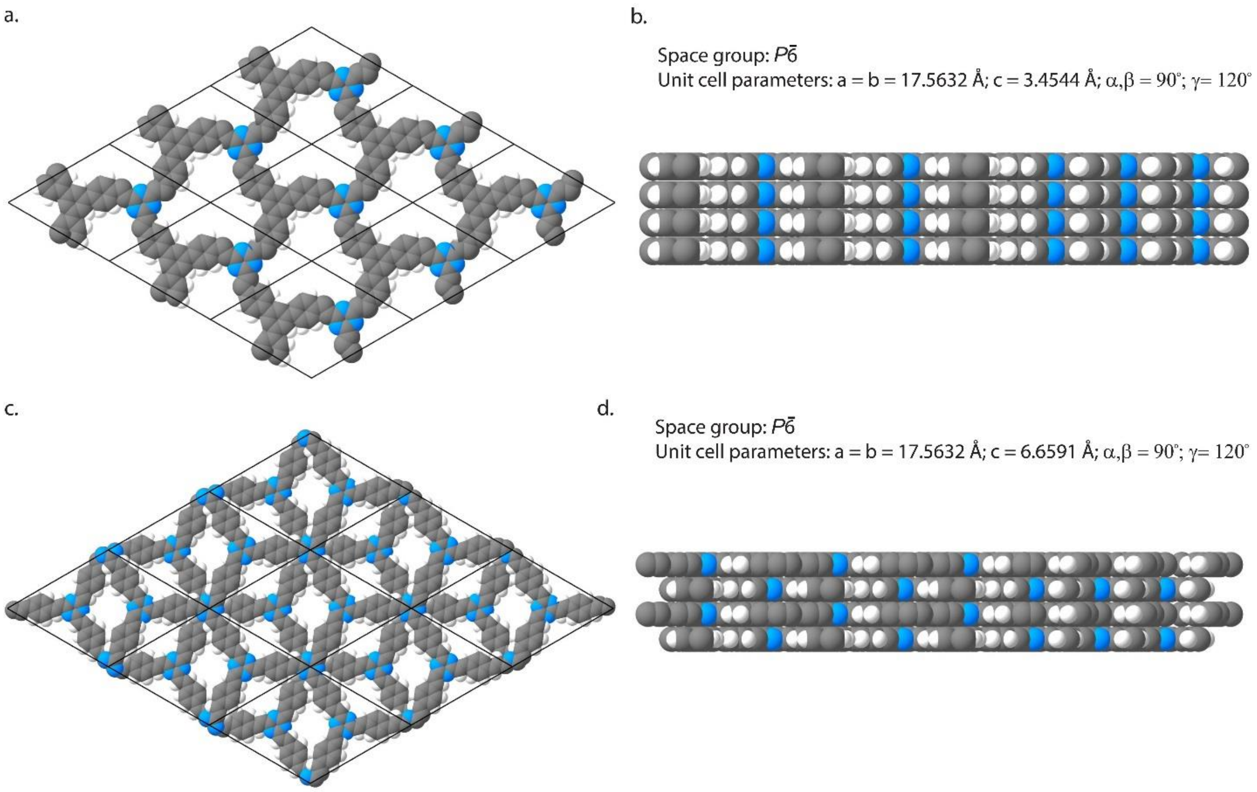


**Figure S4.** Structural models of V-COF-2. a) Theoretical model of V-COF-1 with eclipsed (AA) stacking arrangement. b) Side view of V-COF-2 structure with eclipsed stacking arrangement c) The theoretical model of V-COF-2 with staggered (AB) stacking arrangement. d) Side view of V-COF-2 structure with staggered stacking arrangement.

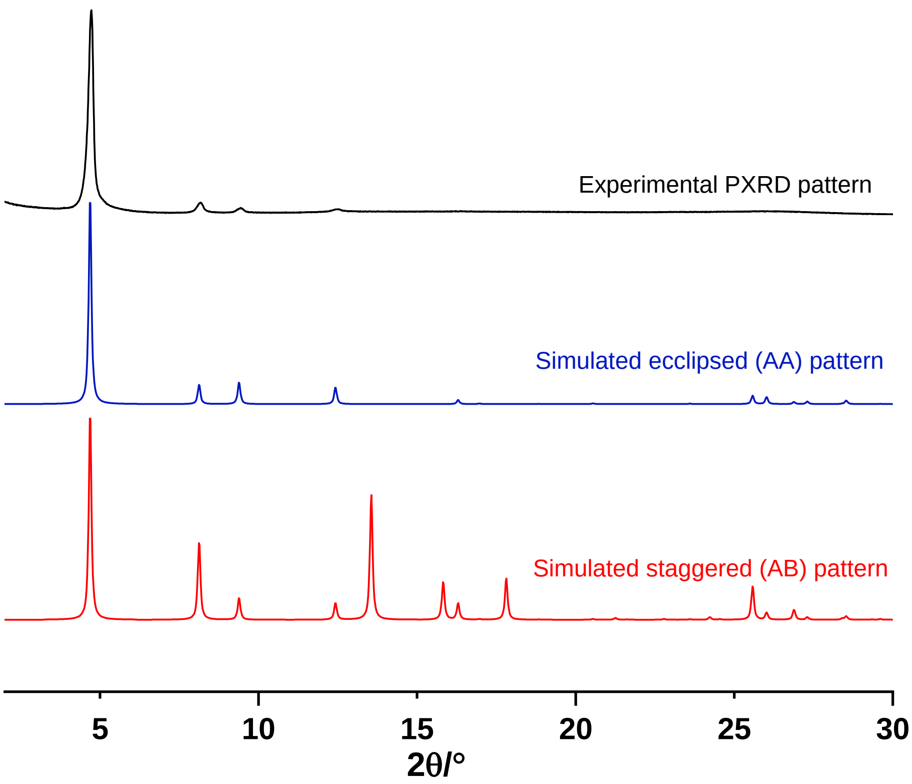


**Figure S5**. Simulated PXRD patterns for the generated **hcb** models with eclipsed (blue) and staggered (red) stacking mode compared to the experimentally obtained pattern of V-COF-1 (black).

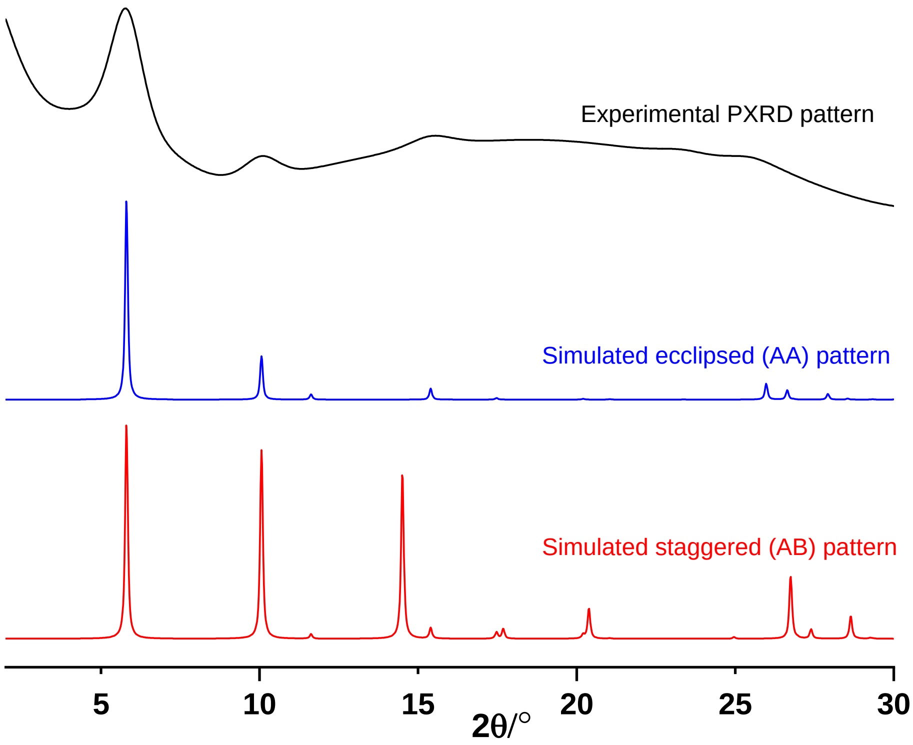


**Figure S6**. Simulated PXRD patterns for the generated **hcb** models with eclipsed (blue) and staggered (red) stacking mode compared to the experimentally obtained pattern of V-COF-2 (black).

**Table S1.** Fractional atomic coordinates for V-COF-1*:*

| ***V-COF-1*** Space group symmetry: *P*6/*m* (175) *a* = *b* = 21.6934 Å; *c* = 3.5232 Å *α* = *β* = 90˚; γ = 120˚ | | | | |
|---|---|---|---|---|
| **Atom Name** | **Atom** | ***x*** | ***y*** | ***z*** |
| C1 | C | 0.2660 | 0.6346 | 0.01409 |
| C2 | C | 0.3986 | 0.5838 | 0.02225 |
| C3 | C | 0.4564 | 0.6014 | 0.01145 |
| C4 | C | 0.4807 | 0.5529 | -0.00219 |
| C5 | C | 0.5523 | 0.5702 | 0.00201 |
| C6 | C | 0.4293 | 0.4810 | -0.00684 |
| N7 | N | 0.3000 | 0.7045 | -0.01938 |
| H8 | H | 0.3815 | 0.4674 | -0.00411 |
| H9 | H | 0.5881 | 0.6175 | 0.02338 |
| H10 | H | 0.4885 | 0.6499 | 0.02067 |
| H11 | H | 0.3672 | 0.5351 | 0.00286 |

**Table S1.** Fractional atomic coordinates for V-COF-2*:*

| ***V-COF-2***<br>Space group symmetry: $P\bar{6}$ (174)<br>$a = b$ = 17.5632 Å; $c$ = 3.4544 Å<br>$\alpha = \beta$ = 90˚; γ = 120˚ | | | | |
|---|---|---|---|---|
| **Atom Name** | **Atom** | ***x*** | ***y*** | ***z*** |
| N1 | N | 0.6837 | 0.4159 | 1.0000 |
| C2 | C | 0.6006 | 0.3478 | 1.0000 |
| C3 | C | -0.7330 | -0.4198 | 1.0000 |
| C5 | C | -0.6468 | -0.4016 | 1.0000 |
| C6 | C | -0.8479 | -0.3737 | 1.0000 |
| C7 | C | -1.0260 | -0.4209 | 1.0000 |
| C8 | C | -1.1202 | -0.4492 | 1.0000 |
| C9 | C | -0.9137 | -0.4618 | 1.0000 |
| C10 | C | -0.9990 | -0.4846 | 1.0000 |
| C11 | C | -1.1656 | -0.5350 | 1.0000 |
| C14 | C | -0.9625 | -0.3323 | 1.0000 |
| C15 | C | -0.8738 | -0.3083 | 1.0000 |
| H17 | H | -0.8285 | -0.2386 | 1.0000 |
| H12 | H | -0.9049 | -0.5180 | 1.0000 |
| H13 | H | -1.0438 | -0.5552 | 1.0000 |
| H16 | H | -0.9813 | -0.2821 | 1.0000 |
| H18 | H | -0.7817 | -0.4865 | 1.0000 |

**Section S5.NMR characterization of model compound:**

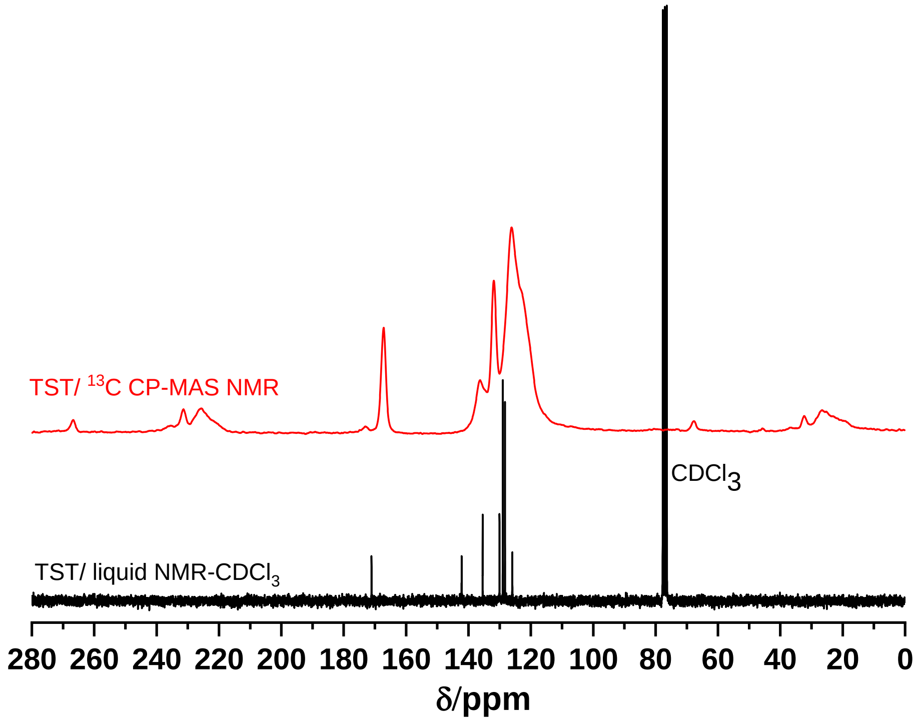


**Figure S7.** Liquid (black curve) and solid state CP-MAS (red curve) $^{13}C$ NMR spectra of TST (model compound).

**Section S6. Pore size distribution analysis of V-COFs:**

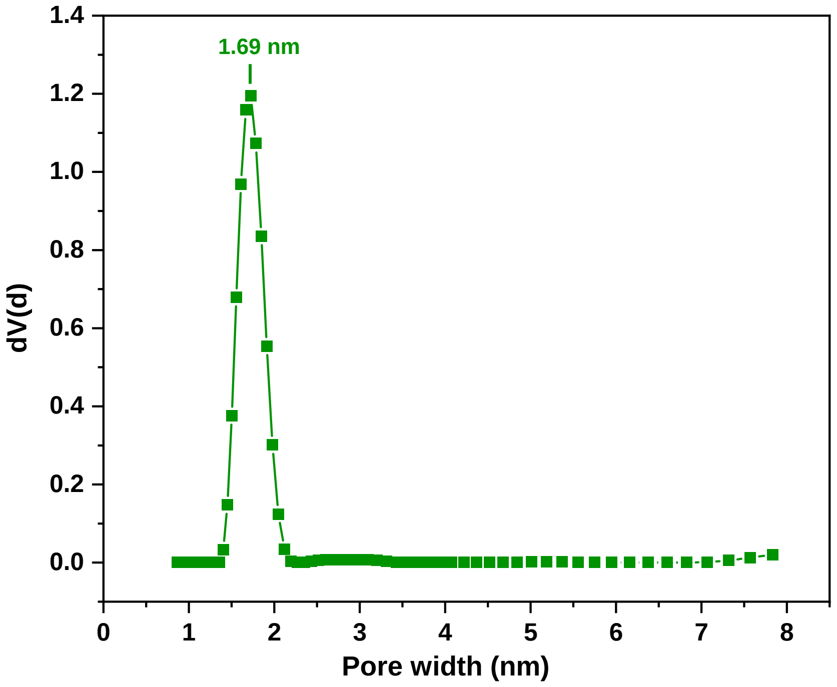


**Figure S8**. Calculated pore size distribution (PSD) plot of V-COF-1 from argon adsorption data at 87K after QSDFT model fitting of adsorption branch data.

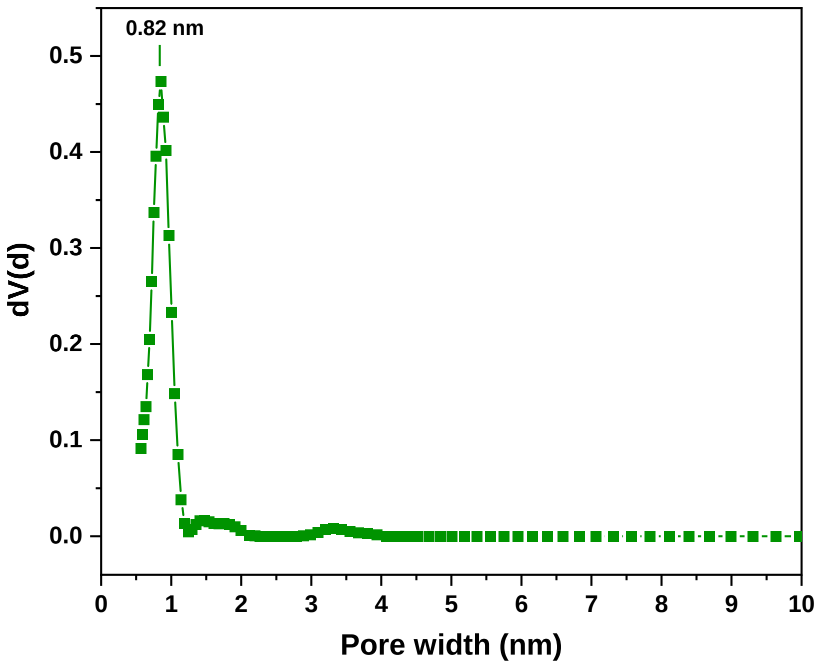


**Figure S9**. Calculated pore size distribution (PSD) plot of V-COF-2 from argon adsorption data at 87K after QSDFT model fitting of adsorption branch data.

**Section S7. Thermo-gravimetric analyses (TGA) of the V-COFs:**

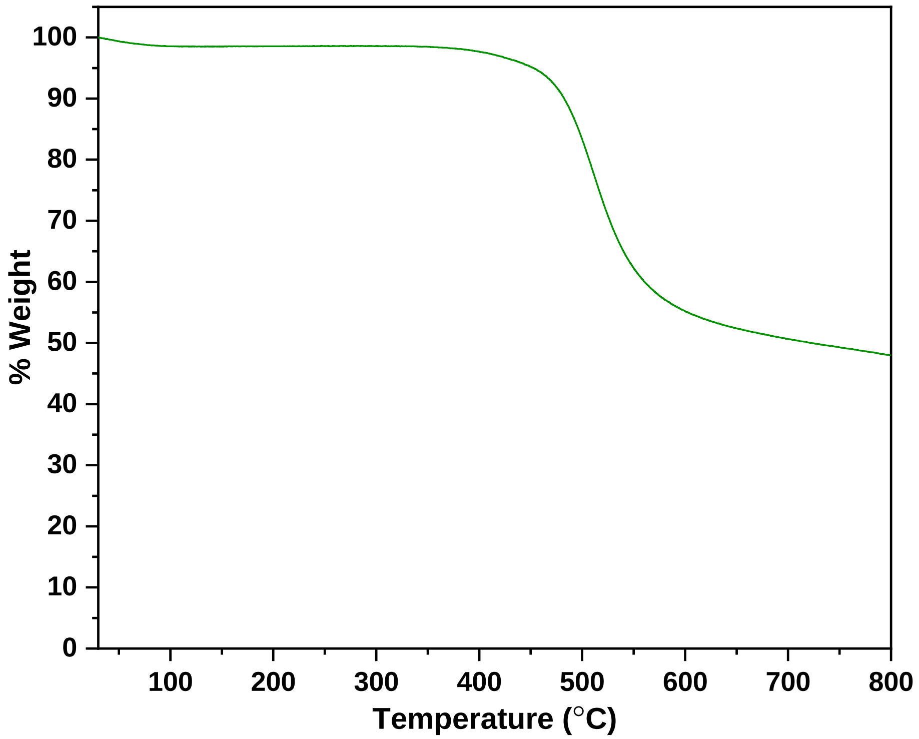


**Figure S10**. Thermo-gravimetric analyses (TGA) of the V-COF-1 under $N_2$ flow.

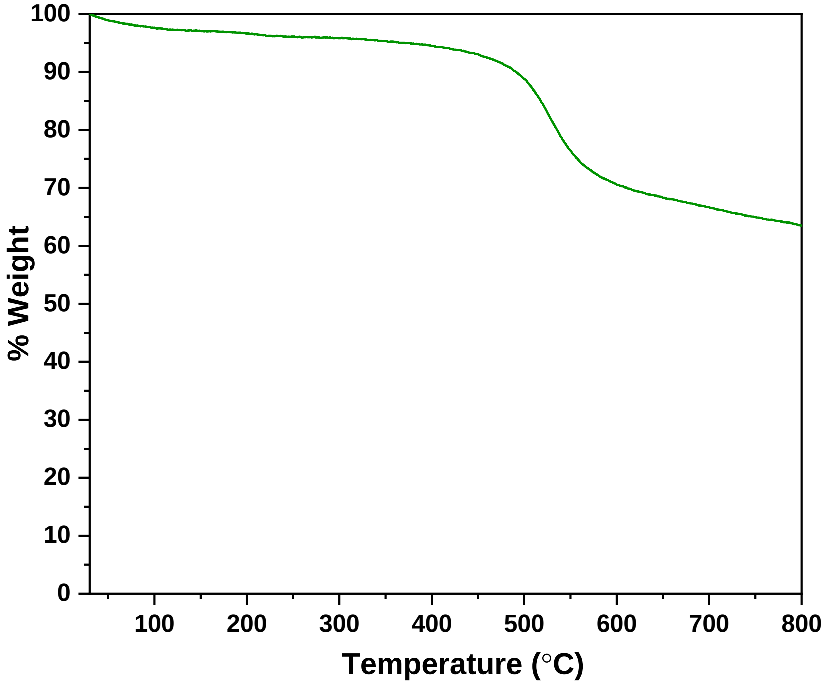


**Figure S11**. Thermogravimetric analyses (TGA) of the V-COF-2 under $N_2$ flow.

**Section S8. Stability test of V-COF-1:**

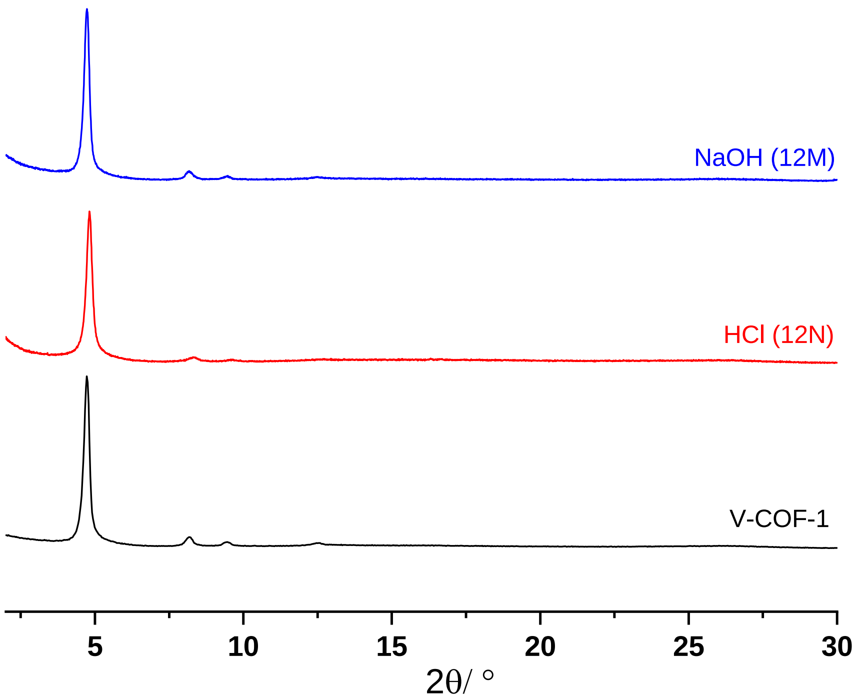


**Figure S12**. PXRD patterns of V-COF-1 after acid (12N HCl) and base (12M NaOH) treatment for 4 days.

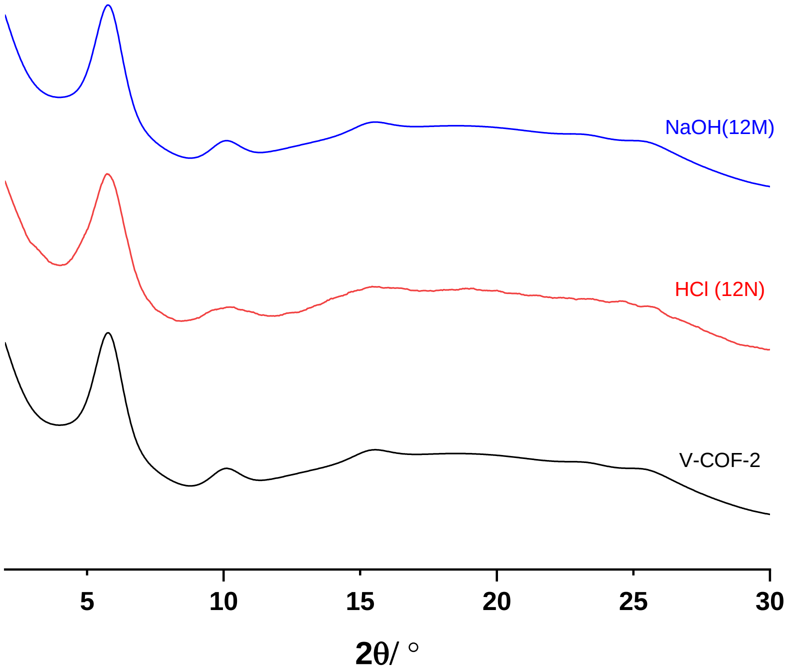


**Figure S13.** PXRD patterns of V-COF-2 after acid (12N HCl) and base (12M NaOH) treatment for 4 days.

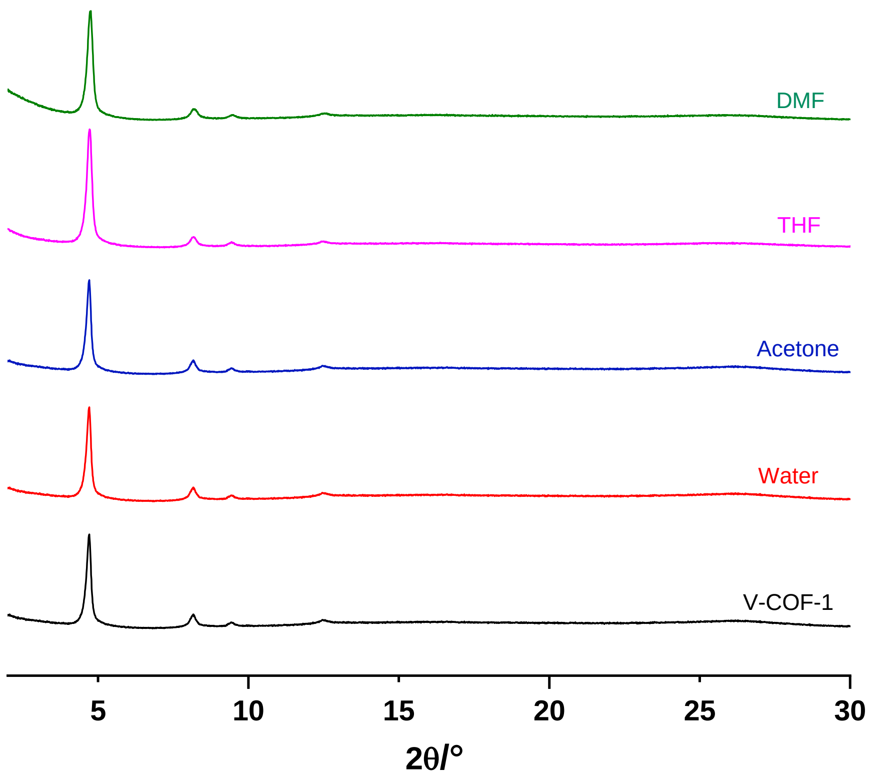


**Figure S14:** PXRD patterns of V-COF-1 after different solvent treatments for 2 days.

**Section S9. Optical properties of V-COF-1:**

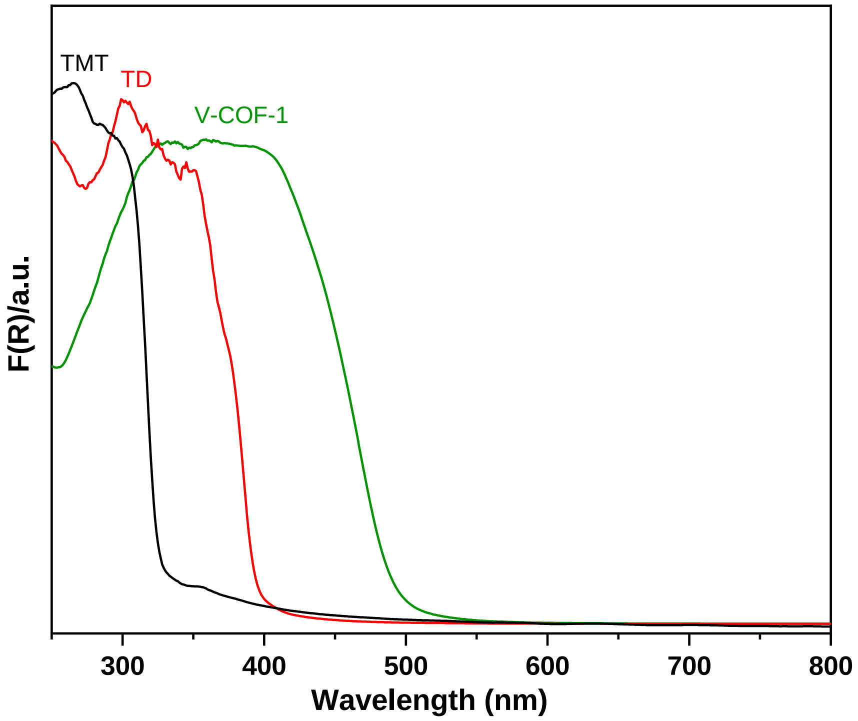


**Figure S15.** Diffuse reflectance UV-Vis spectra of V-COF-1 and starting materials, indicating higher degree of conjugation in V-COF-1.

**Section S10. [2+2]-photocycloaddition:**

**Conditions for [2+2] cycloaddition on V-COF-1 powder:** 50mg of V-COF-1 powder was kept inside a jacketed glass vessel inside a glove box under argon atmosphere. The glass vessel was then sealed with a rubber septum and para film and taken out from the glove box and connected to a water circulation set-up maintaining a temperature of 10 °C. Light (λ~320-500 nm) was irradiated from the bottom to ensure a close distance to the sample. The sample was occasionally shaken to provide a homogeneous light exposure to all parts of the samples.

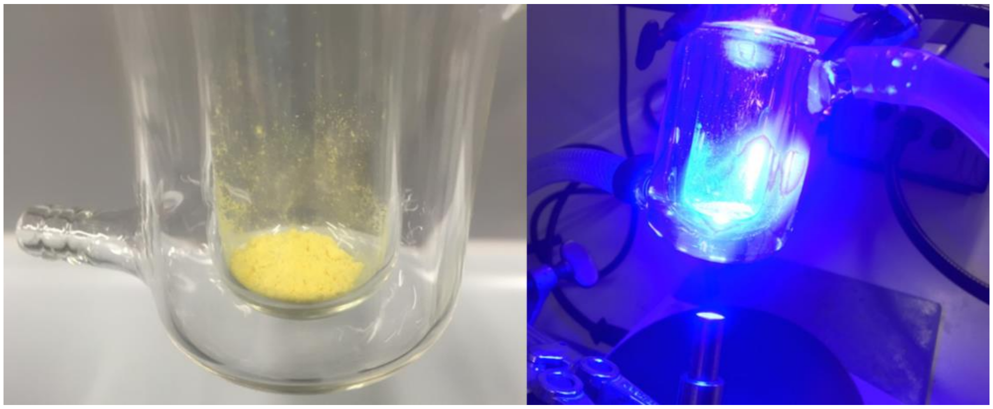

**Figure S16.** Light irradiation set-up for [ 2+2] photocycloaddition on V-COF-1 powders

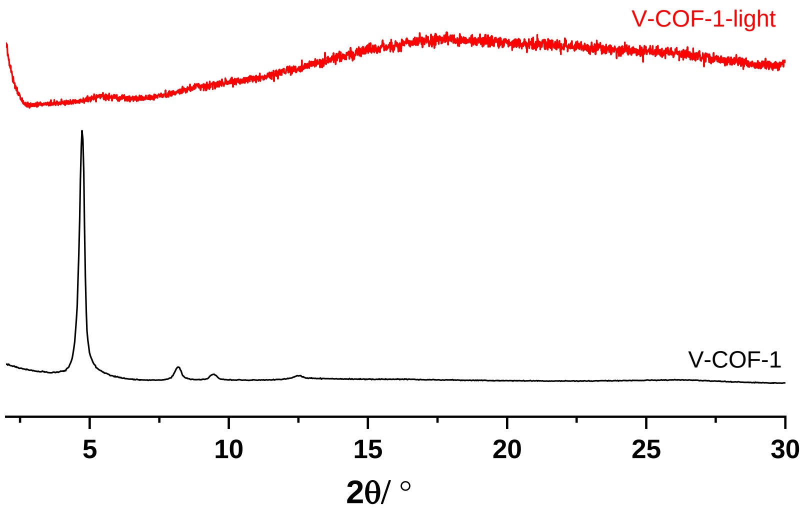


**Figure S17.** Change in crystallinity of V-COF-1 upon [2+2] photocycloaddition leading to an amorphous material (V-COF-1-light).

**References:**


[1] D. N. Bunck, W. R. Dichtel, *J. Am. Chem. Soc.* **2013**, *135*, 14952–14955.
[2] F. C. Schaefer, G. A. Peters, *J. Org. Chem.* **1961**, *26*, 2778–2784.
[3] H. Meier, H. C. Holst, A. Oehlhof, *European J. Org. Chem.* **2003**, *2003*, 4173–4180.
[4] A. S. Accelrys, Material Studio Release Notes, Release 5.0, **2008**.
[5] A. K. Rappe, C. J. Casewit, K. S. Colwell, W. A. Goddard, W. M. Skiff, *J. Am. Chem. Soc.* **1992**, *114*, 10024–10035.
